# Spontaneous rupture of a degenerated leiomyoma causing peritonitis and ileus during pregnancy: A case report

**DOI:** 10.1002/ccr3.3564

**Published:** 2020-11-22

**Authors:** Mizuha Odagami, Mutsuko Makino, Etsuko Miyagi, Shigeru Aoki

**Affiliations:** ^1^ Perinatal Center for Maternity and Neonate Yokohama City University Medical Center Yokohama Japan; ^2^ Department of Obstetrics and Gynecology Yokohama City University Hospital Yokohama Japan

**Keywords:** degenerated leiomyoma, myomectomy, peritonitis, pregnancy, ruptured leiomyoma

## Abstract

Degenerated leiomyomas can rupture during pregnancy, and leakage of the fluid causes peritonitis in rare cases. If peritonitis associated with a degenerated leiomyoma is observed, the occurrence of rupture of the leiomyoma should be considered.

## Graphical abstract

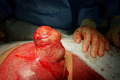

## 1 INTRODUCTION

Leiomyomas are found in approximately 10% of pregnant women.^1^ Pain caused by a degenerated leiomyoma is considered to be the most common complication of pregnancy in women with leiomyomas.^2^ In many cases, the symptoms of a degenerated leiomyoma improve with conservative therapy; thus, surgical treatment is rarely required.^3^, ^4^ We report the case of a 38‐year‐old primiparous woman. She had a ruptured degenerated leiomyoma without abdominal bleeding but showed peritonitis and ileus, which prompted us to perform myomectomy during pregnancy, with a good outcome.

## 2 CASE

A 38‐year‐old primiparous woman was diagnosed with a leiomyoma prior to her pregnancy. The patient had no history of uterine or abdominal surgery. At a gestational age of 14 + 3 weeks, she had lower abdominal pain and was admitted to a primary health facility with a diagnosis of pain due to a degenerated leiomyoma, for which conservative therapy was started. Because of increased abdominal pain, she was transported to our hospital at a gestational age of 15 + 0 weeks. The patient characteristics at the time of hospital presentation were as follows: height, 158 cm; weight, 52 kg; blood pressure, 97/74 mm Hg; pulse rate, 98/min; and body temperature, 37.0°C. On admission, she complained of severe abdominal pain and vomited several times. In addition to the peritoneal irritation sign, a goose egg‐sized mass was palpated in the right upper abdomen, and severe tenderness was observed at the same site. Abdominal ultrasonography revealed an intramural leiomyoma measuring 7 cm in diameter at the uterine fundus. Blood tests showed the following results: white blood cells (WBC), 12300/mm^3^; red blood cells, 411 × 104/mm^3^; hemoglobin, 13.1 g/dL; and C‐reactive protein (CRP), 8.52 mg/dL.

**Figure 1:**
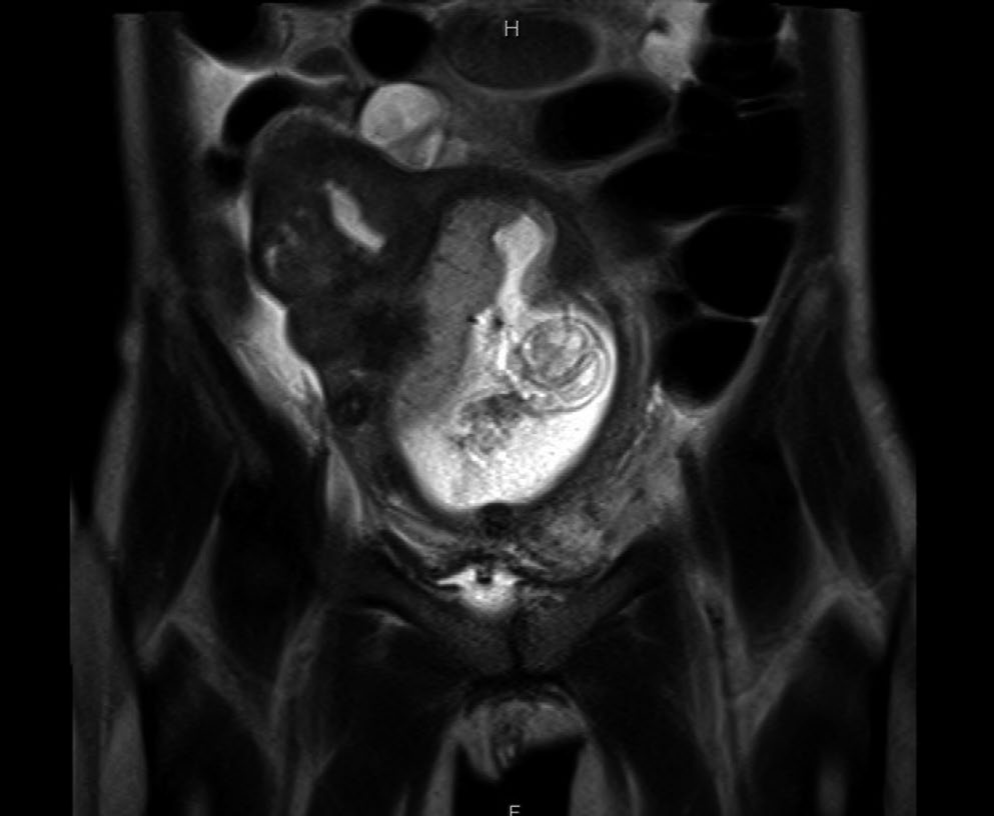
Coronal T2‐weighted magnetic resonance imaging showing a leiomyoma with partial high signal intensity at the uterine fundus, which was considered to be a degenerated leiomyoma

Plain abdominal radiography revealed marked dilatation of the small intestine and ileus was diagnosed. Pelvic magnetic resonance imaging (MRI) revealed a leiomyoma with partial high‐signal intensity at the uterine fundus; it was considered a degenerated leiomyoma (Figure 1). Based on the above findings, the patient was diagnosed with pain from a degenerated leiomyoma, peritonitis associated with infection of the degenerated leiomyoma, and ileus; hence, the conservative therapy was continued. The patient was afebrile after admission and various culture tests, including blood and urine cultures, were negative, indicating no signs of infection. The levels of inflammatory markers decreased after 1 week (WBC: 9600/mm^3^; CRP: 2.3 mg/dL). However, because of the poor improvement in abdominal pain and prolonged ileus, ruptured degenerated myoma was listed as a differential diagnosis, and emergency surgery was performed.

**Figure 2:**
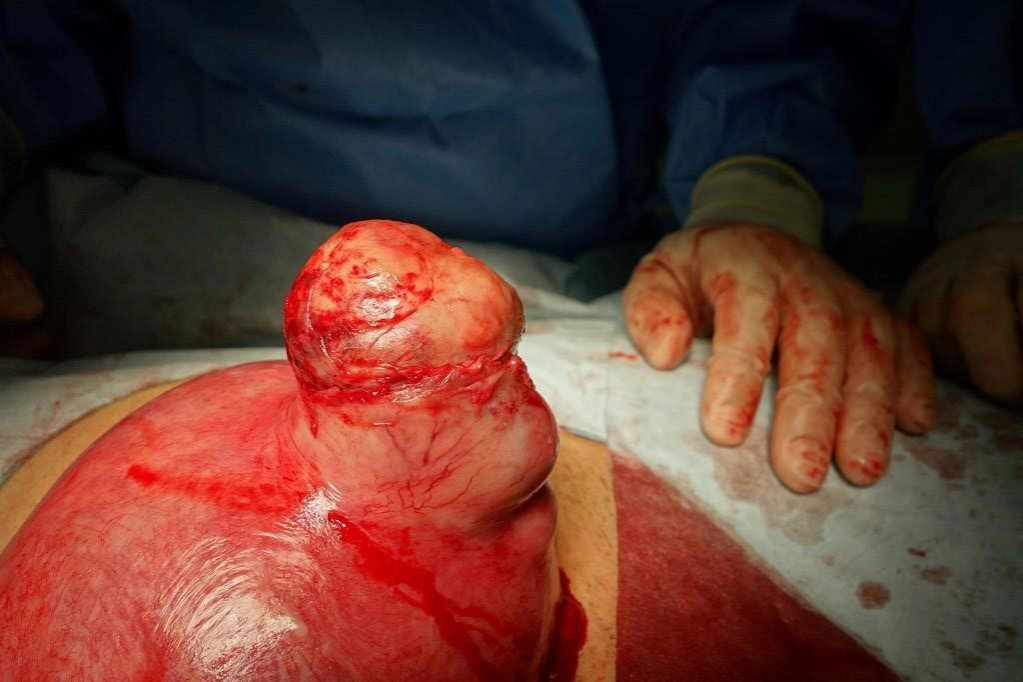
Intraoperative examination revealed an intramural leiomyoma at the uterine fundus, with the upper half of its capsule ruptured

The intraoperative examination revealed an intramural leiomyoma measuring 7 cm in diameter at the uterine fundus. When the omentum and small intestine adhering to the surface of the leiomyoma were dissected, the leiomyoma, with its capsule ruptured, was exposed (Figure 2). Accordingly, the patient was diagnosed with a ruptured, degenerated leiomyoma and a myomectomy was performed. The pathological findings of the resected specimen indicated a leiomyoma with degeneration. After the surgery, the symptoms rapidly improved and the pregnancy course was uneventful. At a gestational age of 37 + 4 weeks, elective cesarean delivery was performed because of the myomectomy during pregnancy. The patient delivered a female infant weighing 2870 g, which was appropriate for the gestational age. The Apgar score was 9 at both 1 and 5 minutes. The postoperative course was uneventful.

## 3 DISCUSSION

This case highlighted the following two findings: First, leiomyomas can rupture during pregnancy. There have been cases reported of the rupture of a blood vessel overlying the leiomyoma during pregnancy^5^, ^6^ and after labor,^7^ resulting in intra‐abdominal bleeding; a case of nonreassuring fetal status and intra‐abdominal bleeding due to the rupture of a leiomyoma during labor has also been reported.^4^ Thus, the rupture of a leiomyoma can occur at any time. The mechanism of leiomyoma rupture has not been clarified. One of the possible mechanisms is that necrosis associated with the degenerated leiomyoma occurs in the uterine serosa and causes a rupture in the serosa. It is also considered that pregnancy, vigorous exercise, and defecation, which increase abdominal pressure, may cause the congestion of a blood vessel overlying the leiomyoma, resulting in its rupture.^8^, ^9^ Therefore, the rupture of a leiomyoma should always be kept in mind when treating a pregnancy complicated by a leiomyoma. Second, as the rupture of a leiomyoma causes peritonitis, it is an indication for prompt surgical treatment. Leiomyoma rupture usually results in intra‐abdominal bleeding due to the rupture of a blood vessel overlying the leiomyoma, often leading to an acute abdomen.^5^, ^6^, ^9^, ^10^ However, according to some reports, the rupture of a degenerated leiomyoma caused no intra‐abdominal bleeding but caused peritonitis due to leakage of the fluid content of the degenerated leiomyoma into the peritoneal cavity.^11^, ^12^ Our case is considered similar to the latter case. In addition to the pain caused by leiomyoma degeneration, if intraperitoneal bleeding, peritonitis, or ileus is present, leiomyoma rupture should be considered. In such cases, prompt diagnosis and surgical treatment are required to avoid an adverse outcome.

## 4 CONCLUSION

Degenerated leiomyoma can rupture during pregnancy. When this occurs, bleeding in the abdominal cavity due to the rupture of blood vessels often results in acute abdomen. In rare cases, however, the fluid from the degenerated leiomyoma leaks into the abdominal cavity, causing peritonitis, and therefore, surgery may be required. In pregnant women with degenerated leiomyomas, we should always keep in mind that leiomyoma rupture may occur.

## 5 AUTHOR CONTRIBUTORS

MO: drafted the manuscript contributed and performed the surgery. MM: performed the surgery. SA: contributed to the first draft and finalization of the manuscript. EM: supervised the case report.

## CONFLICT OF INTEREST

None declared.

## ETHICAL APPROVAL

Prior to submission, appropriate consent for publication of images and data has been obtained.

## Data Availability

Data sharing not applicable to this article as no datasets were generated.
